# Reassessing the Role of Brain Tumor Biopsy in the Era of Advanced Surgical, Molecular, and Imaging Techniques—A Single-Center Experience with Long-Term Follow-Up

**DOI:** 10.3390/jpm11090909

**Published:** 2021-09-12

**Authors:** Rina Di Bonaventura, Nicola Montano, Martina Giordano, Marco Gessi, Simona Gaudino, Alessandro Izzo, Pier Paolo Mattogno, Vittorio Stumpo, Valerio Maria Caccavella, Carolina Giordano, Liverana Lauretti, Cesare Colosimo, Quintino Giorgio D’Alessandris, Roberto Pallini, Alessandro Olivi

**Affiliations:** 1 Department of Neurosurgery, Fondazione Policlinico Universitario “A. Gemelli” IRCCS, Università Cattolica del Sacro Cuore, Largo A. Gemelli, 8, 00168 Roma, Italy; rina.dibonaventura@guest.policlinicogemelli.it (R.D.B.); nicolamontanomd@yahoo.it (N.M.); alessandro.izzo@guest.policlinicogemelli.it (A.I.); pierpaolo.mattogno@policlinicogemelli.it (P.P.M.); vittorio.stumpo@yahoo.it (V.S.); valeriom.caccavella@gmail.com (V.M.C.); liverana.lauretti@policlinicogemelli.it (L.L.); or quintinogiorgio.dalessandris@policlinicogemelli.it (Q.G.D.); Roberto.Pallini@unicatt.it (R.P.); alessandro.olivi@policlinicogemelli.it (A.O.); 2 Department of Pathology, Fondazione Policlinico Universitario “A. Gemelli” IRCCS, Università Cattolica del Sacro Cuore, Largo A. Gemelli, 8, 00168 Roma, Italy; marco.gessi@policlinicogemelli.it; 3 Department of Radiology, Fondazione Policlinico Universitario “A. Gemelli” IRCCS, Università Cattolica del Sacro Cuore, Largo A. Gemelli, 8, 00168 Roma, Italy; simona.gaudino@policlinicogemelli.it (S.G.); carolinagiordano91@gmail.com (C.G.); cesare.colosimo@policlinicogemelli.it (C.C.)

**Keywords:** high-grade glioma, primary brain lymphoma, biopsy, follow-up, radiology

## Abstract

Brain biopsy is the gold standard in order to establish the diagnosis of unresectable brain tumors. Few studies have investigated the long-term outcomes of biopsy patients. The aim of this single-institution-based study was to assess the concordance between radiological and histopathological diagnoses, and the long-term patient outcome. Ninety-three patients who underwent brain biopsy in the last 5 years were analyzed. We included patients treated with stereotactically guided needle, open, and neuroendoscopic biopsies. Most patients (86%) received needle biopsy. Gliomas and primary brain lymphomas comprised 88.2% of cases. The diagnostic yield was 95.7%. Serious complication and death rates were 3.2% and 2.1%, respectively. The concordance rate between radiological and histological diagnoses was 93%. Notably, the positive predictive value of radiological diagnosis of lymphoma was 100%. Biopsy allowed specific treatment in 72% of cases. Disease-related neurological worsening was the main reason that precluded adjuvant treatment. Adjuvant treatment, in turn, was the strongest prognostic factor, since the median overall survival was 11 months with vs. 2 months without treatment (*p* = 0.0002). Finally, advanced molecular evaluations can be obtained on glioma biopsy specimens to provide integrated diagnoses and individually tailored treatments. We conclude that, despite the huge advances in imaging techniques, biopsy is required when an adjuvant treatment is recommended, particularly in gliomas.

## 1. Introduction

The diagnosis of brain tumors relies on the rigorous histopathological assessment of the surgically excised tissue. This is conducted, in the majority of cases, at the time of tumor removal. When this is not possible because of the location of the tumor or because the patient is not amenable to an invasive surgical procedure, a biopsy becomes paramount to characterize the tumor and to offer the proper guidance for subsequent treatments.

Different methods for obtaining a brain biopsy are available. Stereotactically guided needle biopsy is the method of choice for deep-seated lesions and allows adequate tissue sampling while minimizing the surgical risks. Frame-based methods have been the gold standard for decades, while frameless techniques (developed with the advancement of navigational technologies) have been increasingly adopted in the last years, with similar diagnostic yield, morbidity, and mortality [1,2]. In addition, recent improvements in optics, illumination, and micro-camera technologies have prompted the increasing popularity of endoscopic biopsies for intraventricular and paraventricular lesions [3].

Advances in radiological techniques and data processing software have allowed, in recent years, a net gain in the predictive value of diagnostic imaging. Moreover, the identification of biomolecular profiles has increased our ability to characterize these tumors and, at the same time, has opened a new field of molecular imaging (radiomics) [4,5,6].

While many data have been accumulated on the diagnostic value and the safety of different brain biopsy techniques, little is known about the mismatch between pre-operative imaging findings and definitive histopathological diagnoses, and about the long-term follow-up of biopsy patients. The aim of the present study is to report on our series of 93 consecutive biopsy patients, focusing on the reliability of pre-operative radiological diagnosis and on the prognostic factors for survival, in order to reassess the role of brain biopsy in current neuro-oncologic practice.

## 2. Materials and Methods

### 2.1. Patient Selection

We retrospectively reviewed the charts of patients with brain lesions who underwent biopsy at the Department of Neurosurgery of Fondazione Policlinico “A. Gemelli” IRCCS, Rome, between July 2014 and June 2019 (Supplementary Digital Material 1: Supplementary Table S1 online content only). All patients signed an informed consent form according to the research proposals of the Ethical Committee. Age, sex, tumor location, Karnofsky performance status (KPS), pre-operative diagnosis based on MRI report, type of biopsy, surgical complications, length of hospital stay, operation time, histology, and adjuvant treatments (radiotherapy and/or chemotherapy, steroids or other anti-inflammatory drugs) were recorded. Survival was defined as the time interval between biopsy and death from any cause. Survival data were censored in December 2019.

### 2.2. Radiological Evaluation

**Figure 1:**
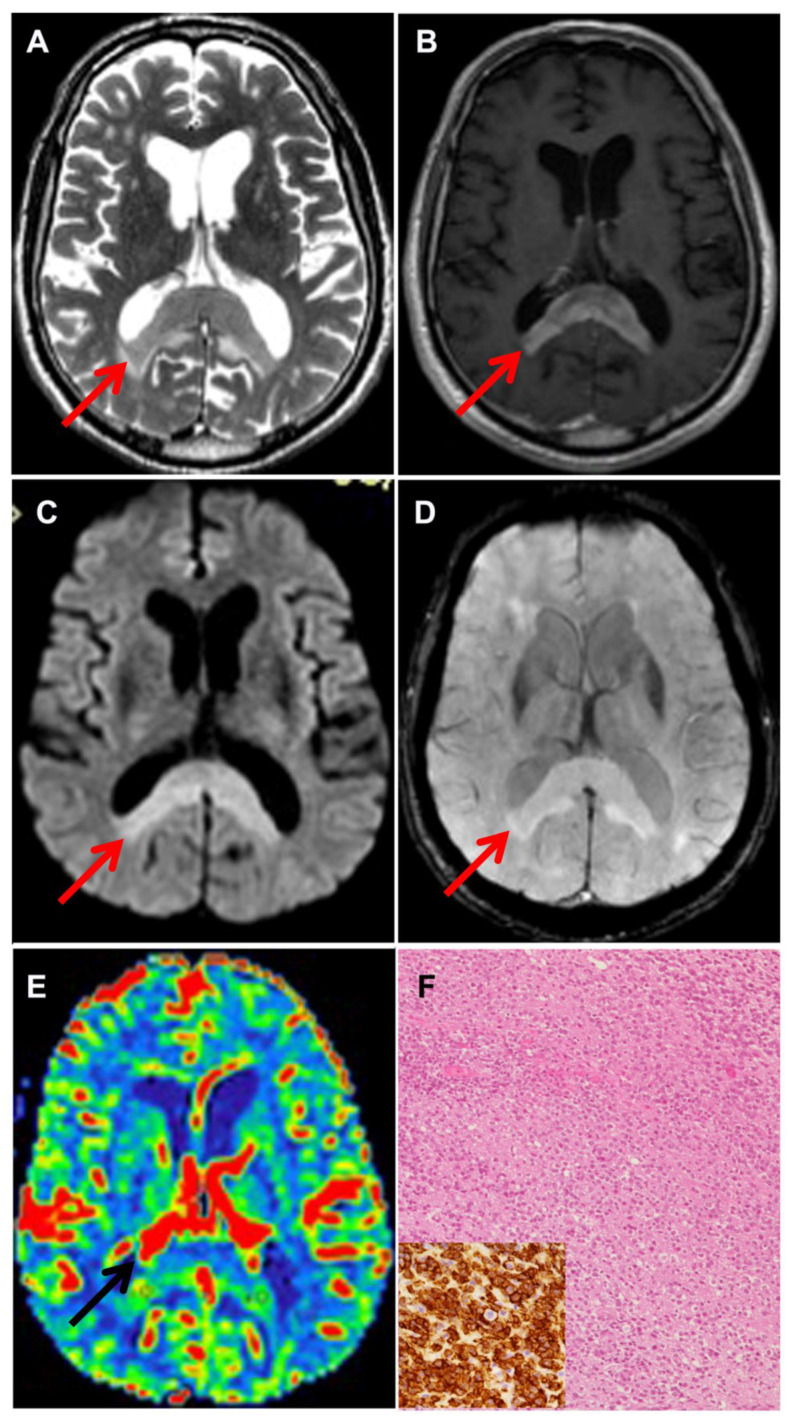
Neuroradiological and histological features of PCNSL. (**A**) Axial T2-weighted and (**B**) axial Gd-enhanced T1-weighted MR imaging showing a T2 hypointense mass involving splenium of the corpus callosum, without necrosis, and with vivid contrast enhancement. DWI (**C**) showing restricted diffusion and SWI (**D**) showing absence of intratumoral hemorrhage. Arrows point at the tumor (**A**–**E**). CBV map (**E**) shows moderately increased intratumoral rCBV. The neuropathological examination reveals a high-grade diffuse B cell non-Hodgkin lymphoma (**F**) showing strong CD20 positivity (**F**, inset).

**Figure 2:**
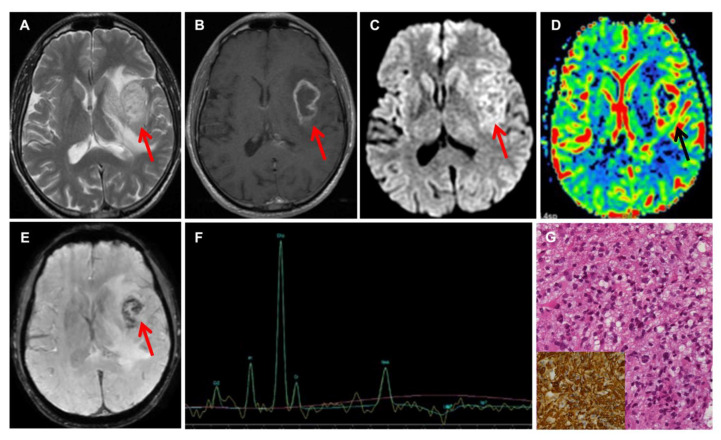
Neuroradiological and histological features of HGG. Axial T2-weighted (**A**) and axial Gd-enhanced T1-weighted (**B**) MR imaging showing a heterogeneous mass in the left insula and basal ganglia, with irregular ring enhancement, corresponding to areas of restricted diffusion on DWI (**C**) and elevated perfusion on CBV map (**D**). SWI (**E**) shows extensive “blooming” foci, consistent with intratumoral hemorrhage. Arrows point at the tumor (**A**–**E**). MRS (**F**) shows elevated Cho and decreased NAA. MRI features are consistent with GBM. The neuropathological examination confirms the presence of a high-grade diffuse astrocytic glioma (**G**) with evidence of vascular proliferation (not shown). The tumor is *IDH*-wt and shows strong immunoreactivity for GFAP (**G**, inset).

All patients underwent a pre-operative magnetic resonance imaging (MRI) with morphological and non-morphological sequences on 1.5 T machine scanners (Signa Excite and EchoSpeed; GE Healthcare, Milwaukee, Wisconsin, USA; Achieva, Philips Healthcare, Best, the Netherlands). Conventional MRI has a limited ability of discriminating adult brain tumors; therefore, advanced MRI sequences, such as DWI, SWI, and perfusion (DSC), were part of our protocol. In selected cases, MR spectroscopy was also included. We measured the maximum rCBV value both in the tumor and in the peritumoral edema, which was defined as the brain tissue with high signal intensity on the FLAIR sequence and with no contrast enhancement on T1W images after Gd administration. We obtained the tissue signal intensity time curves (TSITC) and the percentage of signal recovery (PSR). In each tumor, the apparent diffusion coefficient (ADC) was derived from the DWI, and intratumoral susceptibility signal intensity (ITSS) measured on SWI was also evaluated. The pre-operative radiological differential diagnosis between high-grade glioma (HGG) and primary central nervous system lymphoma (PCNSL) was formulated by combining MRI data obtained by perfusion, diffusion, and susceptibility images [7]. With respect to HGG, PCNSL shows lower signal on T2-weighted images, less necrosis, more intense contrast enhancement, lower rCBV values, higher PSR, T1-dominant leakage pattern, and low ADC within the solid part of the tumor (Figure 1 and Figure 2) [7,8,9,10].

### 2.3. Indication for Biopsy

Indication for biopsy was established by the institutional multidisciplinary neuro-oncology team. Major indications for biopsy were as follows: (1) multifocal or diffusely infiltrating tumors not candidate for debulking surgery; (2) deep-seated tumors not amenable to safe surgical resection; (3) elderly patients harboring large tumors (either multi-centric or deep-seated); and (4) cases with radiological suspicion of PCNSL.

### 2.4. Type of Biopsy

Three surgical biopsy techniques were used (Table 1). Needle biopsy was performed via a burr hole using a dedicated frameless system (StealthStation, Medtronic, Minneapolis, MN, USA). Open biopsy was performed via a mini-craniotomy with standard microsurgical technique.

Neuroendoscopic biopsies for tumors located within or in close proximity with the ventricular system were performed via a burr hole using a ventricular neuroendoscope (Decq endoscope, Karl Storz, Tuttingen, Germany) with a 30° lens. As per standard procedure, three to six samples of the tumor were taken 4 mm apart, and one of them was sent for a frozen histological examination to rule out non-pathological tissue.

### 2.5. Statistical Analysis

Comparison of categorical variables was performed with the chi-square statistic, using the Fisher exact test when appropriate. Median follow-up was calculated using the reverse Kaplan–Meier method. For survival analysis, Kaplan–Meier curves were plotted and compared using the log-rank test. A multivariate analysis for predictive factors for overall survival was performed in the subgroup of the HGG and PCNSL patients treated with needle biopsy, using the Cox proportional hazards model, while adjusting for age, KPS, tumor location, histology, surgical complications, and adjuvant therapy. StatView ver. 5.0 software (SAS Institute, Cary, NC, USA) was used for all analyses. *p* < 0.05 was considered statistically significant.

## 3. Results

### 3.1. Baseline Characteristics of Patients

Ninety-three patients underwent brain biopsy (Supplementary Digital Material 1: Supplementary Table S1 online content only). Median age was 64 years; most patients (88%) were independent or almost independent (KPS ≥ 60) in activities of daily life. Median follow-up was 29 months.

### 3.2. Surgery and Diagnosis

Overall, 94 surgical procedures were completed (Table 1). One patient underwent two biopsies. The majority of procedures (*n* = 80, 85.1%) were performed with the frameless stereotactic (needle) technique. Gliomas and PCNSLs made up 87.2% of cases (Figure 1 and Figure 2). In four cases, a definitive histopathological diagnosis could not be established by a needle biopsy. In one of the cases, a patient who had undergone a short cycle of steroid therapy at another institution, the histological examination of the needle biopsy specimen showed a non-specific lymphocytic infiltrate (Supplementary Digital Material 2: Supplementary Figure S1 online content only). Due to the strong radiological suspicion of PCNSL, biopsy with the open technique was repeated, thus confirming the radiological diagnosis of PCNSL (Supplementary Digital Material 2: Supplementary Figure S1 online content only). The second patient harbored a bilateral inferior frontal mass lesion with a radiological suspicion for low-grade glioma. Needle biopsy resulted in non-diagnostic findings. At craniotomy, a diagnosis of low-grade glioma was obtained. The patient did not undergo any adjuvant treatment and was recurrence-free at the 45-month follow-up. In the two other cases, histological examination showed brain tissue without evidence of a pathological process. By repeating the MRI and CSF analysis, one patient was later diagnosed with demyelinating disease. Both of them are alive after 60–72 months with no signs of progression of the disease.

Interestingly, MGMT promoter methylation was tested in 39 needle biopsy cases of HGG, using methylation-specific PCR [11], and could be determined in all but 3 cases (92.3%). The MGMT promoter was methylated in 61.1% and unmethylated in 38.9% of these cases. In 29 patients, further molecular profiling, including RT-PCR for EGFRvIII and immunohistochemistry for VEGF, was performed [12]. Thus, needle biopsy is an invaluable tool to assess diagnosis, particularly in the era of molecularly tailored, precision medicine.

Surgical complications occurred in eight cases (8.6%). Post-operative brain edema was recorded in three patients, resulting in severe neurological impairment leading to death in two of the cases (2.1%), while the third patient experienced a post-operative hemiparesis. Seizures were recorded in two cases and pulmonary embolism in three patients. Notably, no clinically significant post-operative hemorrhages were reported (Table 1).

### 3.3. Correlation between Pre-Operative MRIs and Biopsy Histology

Overall, a pre-operative radiological diagnosis was made in 90% (82/91) of cases (Table 2). The two patients without definitive histological diagnosis were excluded from this analysis. Agreement between pre-operative radiological diagnosis and histology was achieved in 93% of cases (Table 2).

Among the 17 histologically confirmed PCNSL cases, 11 (64.7%) had a pre-operative radiological diagnosis of PCNSL, 1 (5.9%) of HGG, and 5 (29.4%) an uncertain pre-operative diagnosis. All cases (11/11) with a pre-operative radiological diagnosis of PCNSL had this diagnosis confirmed by histology; therefore, the positive predictive value of radiological diagnosis of PCNSL was 100% in our series (Table 2). Among the 61 histologically confirmed HGG cases, 60 (98.4%) had a pre-operative radiological diagnosis of HGG, while 1 case (1.7%) had an uncertain diagnosis. Out of 64 patients with a pre-operative radiological diagnosis of HGG, 60 (93.8%) were confirmed on histology.

In nine patients (10%), a pre-operative radiological diagnosis could not be made (Table 2). In six of them, the issue between HGG and PCNSL could not be addressed by radiology. In these cases, histology revealed PCNSL in five cases and HGG in one case. The other three patients harbored metastasis, pilocytic astrocytoma of diencephalon, and inflammatory lesion.

### 3.4. Follow-Up of Biopsy Patients

A total of 72% of HGG patients and 76.5% of PCNSL patients underwent adjuvant therapy after biopsy (Table 3). Twenty-one patients harboring HGG or PCNSL did not receive adjuvant therapy after biopsy. The main reason for not undergoing adjuvant therapy was a rapid neurological worsening due to tumor progression (11/21, 52.4%). Other reasons were surgical complications (*n* = 3) and the patient’s decision (*n* = 7). Notably, neither age (<70 years vs. ≥70) nor KPS (<70 vs. ≥70) significantly impacted adjuvant therapy (Fisher exact test).

At the last follow-up, 29% of patients were alive and 71% were dead. Median overall survival was 5.5 months among HGG patients and 5 months among PCNSL patients. As expected, OS was deeply influenced by adjuvant treatment. Median OS was 7.5 and 19 months among the HGG and PCNSL patients who received adjuvant therapy, respectively, and 2 and 1 months among the HGG and PCNSL patients who did not receive adjuvant therapy, respectively (*p* < 0.0001 for HGG and *p* = 0.0008 for PCNSL; log-rank test; Table 3). The PCNSL patients who underwent adjuvant therapy had the best prognosis (Supplementary Digital Material 2: Supplementary Figure S2 online content only).

In the HGG and PCNSL patients diagnosed with needle biopsy (*n* = 69), adjuvant therapy emerged as the only independent predictive factor for OS (*p* < 0.0001 both on univariate and multivariate analyses). There was a non-significant trend of improved prognosis in patients with good pre-operative performance status, thus highlighting the value of a rigorous selection of patients for biopsy surgery. Of note, age was not significantly correlated with OS in this cohort (Supplementary Digital Material 1: Supplementary Table S1 online content only).

## 4. Discussion

In this work, in order to reassess the role of brain biopsy in current neuro-oncologic practice, we specifically addressed (i) the correlation between pre-operative radiological diagnosis and histology and (ii) the long-term outcome of patients undergoing brain biopsy. In addition to the well-established conclusions regarding the safety and reliability of brain biopsy, which are extensively reported on in other studies [1,2], two major findings arise from our study. The first one concerns the accuracy of the radiological diagnosis of PCNSL that, when obtained pre-operatively, was invariably confirmed by histology in all cases. The second finding concerns the outcome of biopsy patients, in whom a significantly prolonged survival is understandably associated with the implementation of the subsequent adjuvant therapies, especially for PCNSL cases. Another important finding, considering the recent development of precision medicine concepts, is the ability to obtain an accurate molecular profiling of CNS tumor patients through needle biopsy.

### 4.1. State-of-Art of Brain Biopsy

The methods of sampling brain and tumor tissue for diagnosis have evolved from explorative craniotomy to frame-based stereotactic approaches to the more recent frameless techniques based on neuro-navigation [13]. This ignited a debate in the scientific community, with some claiming the superiority of the newer frameless technique, and others defending the classic, frame-based technique [1,13,14,15,16,17,18,19]. A recent meta-analysis found no significant differences between frame-based and frameless biopsy. With both techniques, diagnostic yield was 85–100%, morbidity 1.3–28%, and mortality 1.2–3.9%. The only significant difference was a shorter length of surgery with the frameless technique [2]. Technological refinements in the field of brain biopsy include robot-assisted techniques [20,21,22] and neuro-endoscopic procedures for ventricular and periventricular tumors [3].

However, advances in non-invasive diagnostic tools have markedly improved the reliability of radiological diagnoses. The rise of radiomics and the implementation of “machine learning” algorithms have been of help in this process, allowing a pre-operative distinction between PCNSLs and HGGs. For the latter group of tumors, radiomics is expected to predict molecular profiling [4,5,6,23]. Nevertheless, the reliability of radiological assessment is still considered insufficient, and all guidelines recommend obtaining a histological diagnosis before deciding on therapeutic options [11].

### 4.2. Findings of the Present Study

In our series, the diagnostic yield of biopsy was 95.7%. In four patients (4.3%), the biopsy did not permit histological diagnosis. In two of them, craniotomy was necessary to diagnose PCNSL and low-grade glioma. The other two cases, in whom a diagnosis could not be achieved through biopsy, had an indolent course, suggesting inflammatory or regressive changes. The positive predictive value of pre-operative radiological diagnosis was promising, with a 93% overall rate and a remarkable 100% rate for cases with a radiological diagnosis of PCNSL. However, in 10% of cases, a definite pre-operative radiological diagnosis could not be rendered. As for safety, we reported an 8.6% overall complication rate and a 3.2% serious complication rate, which is in line with literature data. Adjuvant therapy was administered to 73% of patients suffering from HGG or PCNSL, leading to an improved survival especially when a diagnosis of PCNSL was made. The majority of patients who did not undergo adjuvant treatments experienced a rapid neurological worsening due to tumor progression.

In the setting of HGG, tissue sampling also carries the important role of defining the molecular profile of the tumor, which is a required task in order to establish the correct diagnosis, particularly for the upcoming 2021 WHO classification of central nervous system tumors [24]. Moreover, in the era of precision medicine, molecular characterization of HGG can impact adjuvant therapy, particularly in elderly and fragile patients [11,12], or it may be required for enrollment in trials with novel targeted or biological agents. In our series, we obtained enough tissue for molecular analysis in more than 90% of cases using needle biopsy.

Our data, while reinforcing the rationale of biopsy surgery for unresectable brain tumors, raise the question of whether a biopsy could be avoided in very selected cases, when radiology can establish a reliable diagnosis of PCNSL. From a pathological viewpoint, PCNSLs are mainly (up to 95%) high-grade B-cell non-Hodgkin lymphomas for which the standard treatment protocol is not influenced by molecular subtyping [11]. If the radiological criteria applied in our study—which had a 100% positive predictive value for PCNSL diagnosis—were confirmed in well-designed, larger trials, PCNSL patients could become possible candidates to receive chemotherapy or radiotherapy without histological confirmation.

### 4.3. Limitations of the Present Study

The main limitation of the study is the retrospective nature of data collection. The single-institution profile of the study can be regarded as a limitation, although it assures the homogeneity of diagnosis, treatment, and follow-up among patients. Finally, the inclusion of patients treated with different surgical techniques could also be a confounding factor. However, this factor was eliminated in the subgroup of HGG and PCNSL patients treated with needle biopsy.

## 5. Conclusions

The results of the present work confirm that biopsy is the gold standard for safely diagnosing brain tumors that are not amenable to surgical resection. As a result of this rigorous and safe approach of obtaining a definitive diagnosis, it is possible to offer the most appropriate adjuvant treatment to these patients and, consequently, aim at extending their survival, particularly in the upcoming era of precision medicine. Therefore, in current neuro-oncologic practice, biopsy is a key step that cannot be avoided when an adjuvant treatment is required. At the same time, however, advances in imaging techniques are reinforcing the predictive value of pre-operative radiological diagnosis. It could also be possible that, in the future, large trials will allow to define criteria for avoiding biopsy, particularly in selected cases of PCNSL.

## Figures and Tables

**Table 1:** Surgical data of 94 brain biopsy procedures.

Biopsy Technique	
Needle	85.1%
Endoscopic	5.3%
Open	9.6%
Histologic Diagnosis	
HGG	64.9%
IDH wild-type	98.2%
IDH mutated	1.8%
Low-grade glioma NOS	4.3%
PCNSL	18.1%
Inflammatory tissue	4.3%
Other	4.3%
Non-diagnostic	4.3%
Complications	8.6%
Brain edema	3.2%
Seizures	2.2%
Pulmonary embolism	3.2%
Death	2.1%

HGG, high-grade glioma; NOS, not otherwise specified; PCNSL, primary central nervous system lymphoma.

**Table 2:** Agreement between pre-operative suspicion and histology.

Pre-Operative Radiological Diagnosis	*n* * (%)	Agreement (%)
Yes	82 (90.1%)	92.7% (76/82)
HGG	64 (70.3%)	93.8% (60/64)
Low-grade glioma	5 (5.5%)	60% (3/5)
PCNSL	11 (12.1%)	100% (11/11)
Inflammatory tissue	2 (2.2%)	100% (2/2)
No	9 (9.9%)	NA

* In 2 cases, a final histological diagnosis was not established. HGG, high-grade glioma; NA, not applicable; PCNSL, primary central nervous system lymphoma.

**Table 3:** Follow-up.

Histology	Adjuvant Therapy (%)	Median Overall Survival (Months)		
		With Adj. Therapy	Without Adj. Therapy	*p* *
Whole group	72.0	11	2	0.0002
HGG	72.1	7.5	2	<0.0001
PCNSL	76.5	19	1	0.0008
Other	66.7	not reached	not reached	0.3765

Adj, adjuvant; HGG, high-grade glioma; PCNSL, primary central nervous system lymphoma. * Log-rank test.

## Data Availability

Data available on request due to privacy restrictions.

## Supplementary Materials

The following are available online at https://www.mdpi.com/article/10.3390/jpm11090909/s1, Supplementary Table S1. Demographic data of 93 patients operated upon for brain biopsy; Supplementary Table S2. Prognostic factors for overall survival among the HGG and PCNSL patients treated with needle biopsy; Supplementary Figure S1. Neuroradiological and histological features of a non-diagnostic needle biopsy in a PCNSL case; Supplementary Figure S2. Survival curves are according to histology (HGG vs. PCNSL) and adjuvant therapy.

## Congresses

Portions of this work have been presented at the 69° Congress of the Italian Society for Neurosurgery, Rome, October 2020.
