# Supplementary material for: Reassessing the Role of Brain Tumor Biopsy in the Era of Advanced Surgical, Molecular, and Imaging Techniques—A Single-Center Experience with Long-Term Follow-Up
Source: J Pers Med. 2021 Sep 12;11(9):909. doi: 10.3390/jpm11090909 (PMC8472374; doi:10.3390/jpm11090909)
Supplement: Supplementary file 1 [file jpm-11-00909-s001.zip › jpm-1357314-supplementary/Supplementary Digital Material 1.pdf]

Supplementary Table SI. ***Demographic data of 93 patients operated upon for brain biopsy***

n	93
Age (years)	
median	64
range	18-82
Sex	
Male (%)	60.2%
Female (%)	39.8%
Location of the lesion	
deep-seated (%)	71%
diffuse/multicentric (%)	29%
Preoperative KPS	
80-100	35%
60-70	53%
<60	12%
Follow-up (months)	
median	29
range	1-78

Supplementary Table SII. ***Prognostic factors for overall survival among HGG and PCNSL patients treated with needle biopsy***

**Parameter**	**Univariate analysis***		**Multivariate analysis**[#]	
	**median OS** (mos)	***p***	**Hazard Ratio**	***p***
**Age** (<70 vs ≥ 70 years)	4.5 vs 5	0.8791	0.812 (0.448-1.475)	0.4945
**KPS** (≥70 vs <70)	5.5 vs 4	0.1737	0.553 (0.285-1.073)	0.0798
**Site** (deep vs diffuse)	5 vs 4.5	0.3657	0.746 (0.421-1.321)	0.3144
**Histology** (HGG vs PCNSL)	5 vs 4.5	0.4140	1.558 (0.674-3.601)	0.2997
**Complications** (no vs yes)	5 vs 1.5	0.0130	0.481 (0.165-1.407)	0.1813
**Adjuvant therapy** (yes vs no)	6.5 vs 1.5	<0.0001	0.184 (0.093-0.364)	<0.0001

*Table notes: HGG, high-grade glioma; KPS, Karnofsky performance status; OS, overall survival; PCNSL, primary central nervous system lymphoma. *, Kaplan-Meier analysis using log-rank test; [#], Cox analysis.*
