# Supplementary material for: Reassessing the Role of Brain Tumor Biopsy in the Era of Advanced Surgical, Molecular, and Imaging Techniques—A Single-Center Experience with Long-Term Follow-Up
Source: J Pers Med. 2021 Sep 12;11(9):909. doi: 10.3390/jpm11090909 (PMC8472374; doi:10.3390/jpm11090909)
Supplement: Supplementary file 1 [file jpm-11-00909-s001.zip › jpm-1357314-supplementary/Supplementary Digital Material 2.pdf]

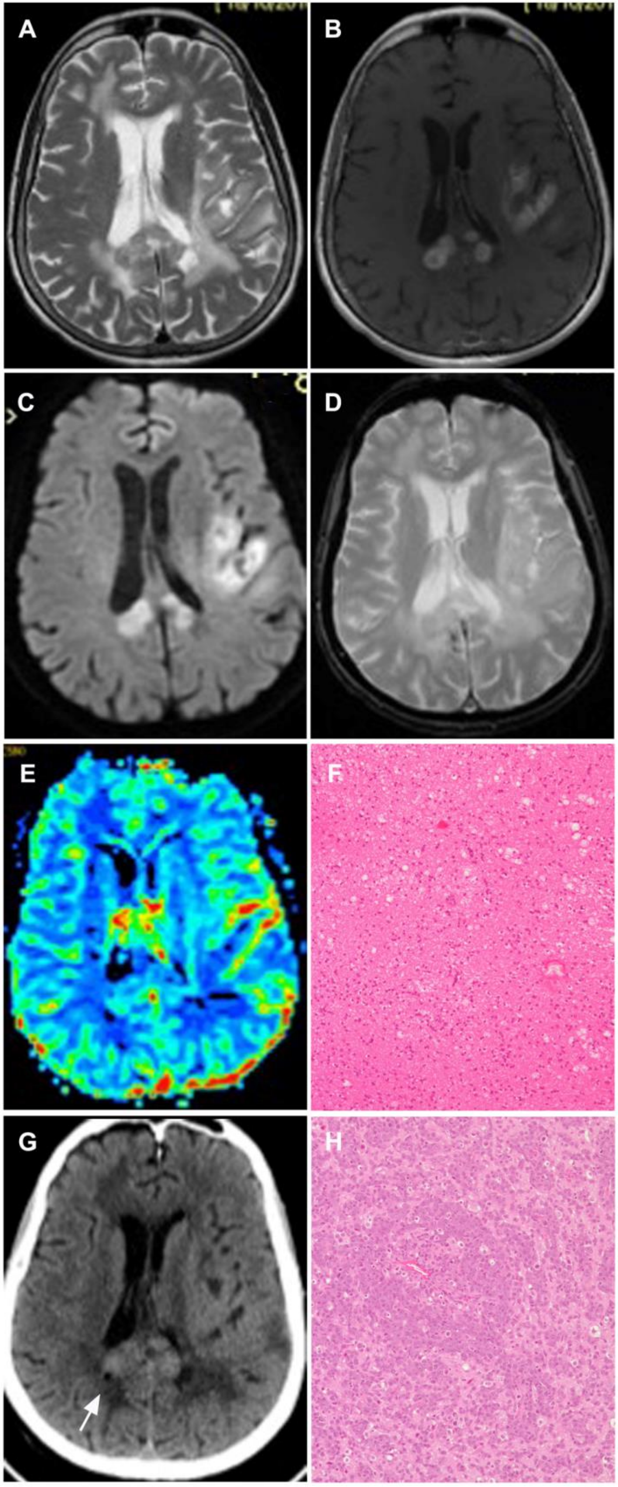

Supplementary Fig. S1 Neuroradiological and histological features of a non-diagnostic needle biopsy in a PCNSL case. Axial T2-weighted (a), axial Gd-enhanced T1-weighted (b) and DWI (c) MR imaging showing multifocal lesions in left insular and parietal lobe and corpus callosum, characterized by relatively low and inhomogeneous T2 signal, marked contrast enhancement, and restricted diffusion. There was no evidence of intratumoral hemorrhage on T2* image (d). CBV map (e) showing moderate higher regional CBV than normal tissue on CBV map; the corresponding perfusion curve (not shown) demonstrated high PSR and a predominant T1 effect. The overall morphological and non-morphological MRI features were highly suggestive for PCNSL. The neuropathological examination of needle biopsy specimen revealed edematous nervous tissue with microglia activation, scant inflammatory infiltrates and reactive gliosis (f) without evidence of lymphoid blasts. Post-operative CT (g) showed air at the biopsy target site (arrow), along the posterior edges of the callosal lesion. The neuropathological specimen obtained via open biopsy displayed a high-grade diffuse B cell non-Hodgkin Lymphoma (h)

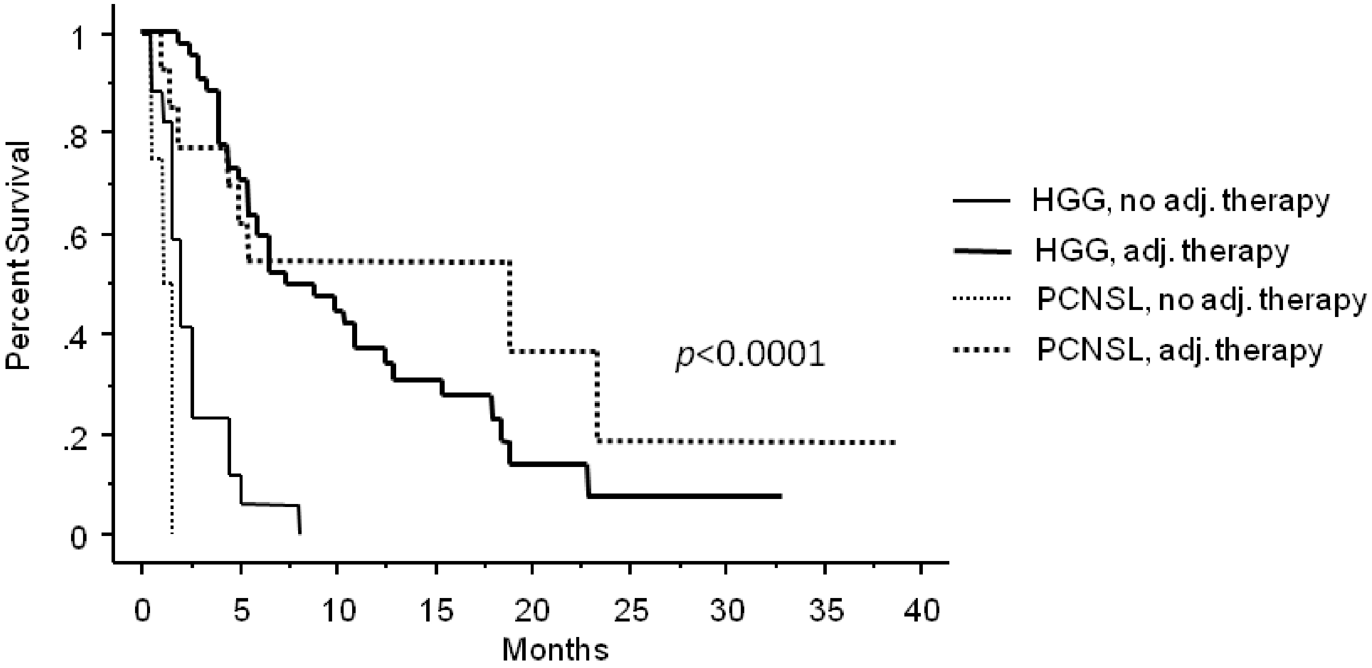

Supplementary Fig. S2 Survival curves according to histology (HGG vs PCNSL) and adjuvant therapy. Adj., adjuvant
